# The Effect of Smoking on Humoral Response to COVID-19 Vaccines: A Systematic Review of Epidemiological Studies

**DOI:** 10.3390/vaccines10020303

**Published:** 2022-02-16

**Authors:** Pietro Ferrara, Vincenza Gianfredi, Venera Tomaselli, Riccardo Polosa

**Affiliations:** 1 Department of Public Health, Experimental and Forensic Medicine, University of Pavia, 27100 Pavia, Italy; p.ferrara5@campus.unimib.it; 2 Center for Public Health Research, University of Milan, Bicocca, 20900 Monza, Italy; 3 Department of Biomedical Sciences for Health, University of Milan, 20133 Milan, Italy; 4 CAPHRI Care and Public Health Research Institute, Maastricht University, 6211 LK Maastricht, The Netherlands; 5 Department of Political and Social Sciences, University of Catania, 95131 Catania, Italy; venera.tomaselli@unict.it; 6 Center of Excellence for the Acceleration of HArm Reduction (CoEHAR), University of Catania, 95131 Catania, Italy; polosa@unict.it; 7 Department of Clinical and Experimental Medicine, University of Catania, 95131 Catania, Italy; 8 Institute of Internal Medicine, AOU “Policlinico-V. Emanuele”, 95131 Catania, Italy

**Keywords:** COVID-19 vaccine, SARS-CoV-2, S-RBD-specific immunoglobulins, smoking, systematic review

## Abstract

While the role of active smoking on response to vaccines is yet to be fully understood, some real-world studies have outlined a possible link between smoking and humoral response to COVID-19 vaccines. Thus, the present rapid systematic review aimed at summarizing the current epidemiological evidence on this association. Following PRISMA and WHO guidelines on rapid systematic reviews, we systematically reviewed published literature on this topic and discussed the findings according to the aim of analysing smoking and its impact on humoral response to COVID-19 postvaccination antibody titres. The search strategy yielded a total of 23 articles. The sample size amongst the studies ranged between 74 and 3475 participants (median, 360), with the proportion of smokers being between 4.2% and 40.8% (median, 26.0%). The studies included in this review analysis investigated the dynamics of antibody response to different type of COVID-19 vaccines. In 17 out of 23 studies, current smokers showed much lower antibody titres or more rapid lowering of the vaccine-induced IgG compared with nonsmokers. This rapid systematic review indicates that active smoking negatively impacts humoral response to COVID-19 vaccines, although the pathophysiologic mechanisms for this association have not been entirely suggested. The results advocate targeted policies to promote tailored health promotion initiatives, which can increase risk perception and ensure appropriate protection measures to be taken to avoid the health consequences of COVID-19 in smokers.

## 1. Introduction

Smoking habit is one of the most common unhealthy behaviours widely prevalent around the globe [1]. Many efforts have been put in place to contrast this habit; however, despite the steady decrement observed in smoking prevalence, the total number of smokers has increased due to the population grown [2,3]. In 2019, more than 1.1 billion tobacco users were censused globally, making smoking one of the most important preventable causes of illness and premature death. In particular, smoking accounts for around 8 million deaths and 200 million disability-adjusted life years each year globally, posing a great challenge to healthcare systems all over the world [3]. The health consequences of smoking include a wide range of illnesses, being a risk factor for lung cancer, chronic obstructive pulmonary disease, cardiovascular diseases, viral and bacterial infections of the respiratory system, and others [4].

Smoking affects the immune system and response. In particular, there is evidence of the association between cigarette smoking and higher risk of several immunological diseases, which range from autoimmune diseases (such as allergies or transplant rejection) to systemic inflammatory diseases (e.g., rheumatoid arthritis), to a lower protection against external antigens, impairing the immunological response to infections [5,6,7,8]. As part of undertakings exploring the impact of cigarette smoking on the immune system, effects on the humoral response after immunization and maintenance of protection induced by several vaccines have been also investigated. Some studies described a link between active smoking and lower levels of vaccine-induced antibodies (such as after immunisation against hepatitis B, and boosters of tetanus and diphtheria) [9,10,11], or increased odds of low-avidity immunoglobulins G (IgG) in smokers (in case of the adjuvanted human papillomavirus type 16 and 18 vaccine) [12]. In contrast, another study on influenza vaccination suggested that smoking does not interfere with the quantity of vaccine-induced antibodies [13]. However, while the effect of cigarette smoking on the humoral response after immunization is generally accepted, the current evidence does not seem to be reliable enough to draw firm conclusions or to generate a consensus, likely due to differences according to vaccine types or in study populations—for instance, in terms of age, comorbidities, and smoking exposure—across the studies. Only limited specific information is available about seroconversion after COVID-19 vaccination in smokers.

With the rapid global diffusion of the coronavirus disease 2019 (COVID-19)—the disease due to the severe acute respiratory syndrome-coronavirus 2 (SARS-CoV-2) [14,15], research efforts have been focused on the swift development of treatments and vaccines. Several COVID-19 vaccines have been developed and authorized since December 2020, and effective immunologic response to after immunization is crucial to limit the negative health outcomes of the pandemic [16,17]. Along with immunization efforts, real-world data have been collected worldwide in order to confirm the safety, immunogenicity, and efficacy of COVID-19 vaccines, and to understand the impact of societal and health factors that might affect the massive vaccination campaigns [17,18]. Some of these analyses have described that vaccine-induced antibody titres are lower or decrease faster among smokers compared with nonsmokers, offering suggestions for further research about the impact of smoking on the humoral response to COVID-19 vaccines [19,20].

In light of the above and the prevalence of smokers, and considering the crucial role of vaccines against COVID-19, we conducted the present systematic review with the objective of summarizing the real-world data from epidemiological studies investigating the impact of smoking and on the humoral response after COVID-19 vaccination.

## 2. Materials and Methods

We carried out a rapid systematic review of evidence on COVID-19 following the World Health Organization (WHO) guidelines: “Rapid reviews to strengthen health policy and systems: a practical guide” [21], and the findings were reported according to the Preferred Reporting Items for Systematic Reviews and Meta-Analyses (PRISMA) guidelines 2020 [22]. Indeed, COVID-19 requires solid and up-to-date evidence to support decision- and policy-making in circumstances of emergency. In this context, the WHO defines a rapid review as a timely and affordable tool that can provide actionable and relevant evidence to strengthen health policy and system research, and promotes its use as practical and suitable approach for collecting and synthetizing growing evidence that can be easily and promptly used to inform stakeholders [21,23].

We surfed PubMed/Medline, Scopus, and Embase databases to retrieve relevant literature. In order to identify eligible articles and documents the lists of references of the included studies were manually screened, and the medRxiv preprints platform and the webpages of international health authorities (including WHO, Centers for Disease Control and Prevention—CDC, and European Centre for Disease Prevention and Control—ECDC) were also consulted. The databases were trawled from inception to December 31, 2021, exploring evidence published during 2021. Only original reports published in English were considered eligible. Our search terms comprised two main aspects, namely COVID-19 vaccine and smoking. The full search string—developed using controlled vocabulary (e.g., MeSH terms) and free-text keywords—is: (“COVID-19 Vaccines”[Mesh] OR “COVID-19 Vaccines”[TIAB] OR ((“COVID-19”[Mesh] OR “COVID-19” OR “SARS-CoV-2”[Mesh] OR “SARS-CoV-2”[TIAB]) AND (“Vaccines”[Mesh] OR “Vaccination”[Mesh] OR vaccin*[TIAB])) AND (“Smokers”[Mesh] OR “Ex-Smokers”[Mesh] OR “Non-Smokers”[Mesh] OR smoker*[TIAB] OR nonsmoker*[TIAB] OR exsmoker*[TIAB] OR smoking[TIAB] OR tobacco[TIAB] OR “heat-not-burn”[TIAB] OR “e-cigarette*”[TIAB] OR “e-cig*”[TIAB] OR “nicotine”[TIAB])). The search strategy was adjusted slightly according to the evaluated databases. Retrieved reports were first evaluated based on title and abstract and only eligible papers were evaluated in full by two authors (PF and VG). In order to be included in the systematic review, papers must fulfil the following criteria: (i) full-text accessible primary epidemiological studies; (ii) reporting immunogenicity data after immunization with any available COVID-19 vaccine; (iii) including smokers. Records that met the following criteria were excluded: (i) studies without data on humoral response to COVID-19 vaccines, (ii) not considering smoking as predictors for vaccine-induced antibody dynamics, (iii) published as review, case report, conference abstract, editorial or letter to editor; (iv) published in language other than English. Data extraction was performed using a prepiloted spreadsheet elaborated in Microsoft Excel^®^ for Windows (Microsoft Corporation, Redmond, WA, USA).

Due to the lack of comparable outcome measures, variability in time points for blood sampling, and quite high methodological heterogeneity across the reports, the results were not pooled in a meta-analysis, but discussed according to the aim to analyse smoking and its impact on humoral response to COVID-19 postvaccination antibody titres.

We also assessed the methodological quality of the body of found evidence through the Grading of Recommendation Assessment, Development and Evaluation (GRADE) guidelines [24]. The assessment of risk of bias across studies was based on the following conditions: (i) representativeness, (ii) selection bias, (iii) reporting bias, (iv) laboratory confirmation of humoral response after vaccination, (v) time between vaccination and sampling, and (vi) proportion of smokers.

## 3. Results

**Figure 1:**
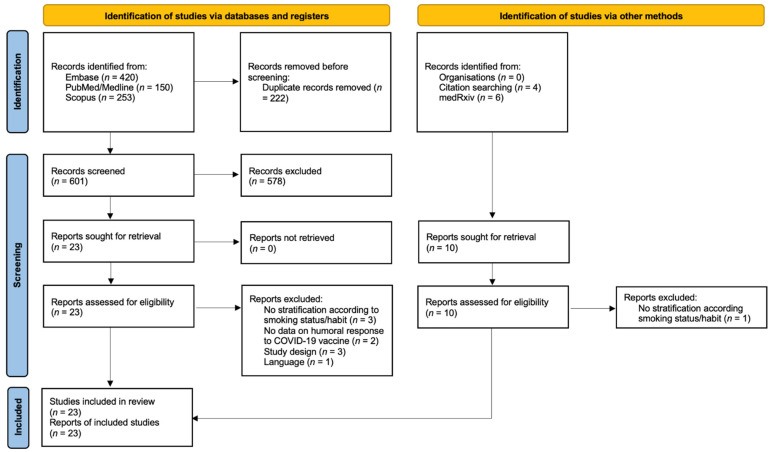
PRISMA flow chart of included studies and selection process.

The flow chart of included studies and selection process is presented in Figure 1.

The search strategy yielded a total of 833 articles. After the reading of the titles and abstracts, and the detection of those that met the exclusion criteria (Supplementary Materials, Table S1), 23 were selected for inclusion in this rapid systematic review, 17 being scientific articles and six being preprints [19,20,25,26,27,28,29,30,31,32,33,34,35,36,37,38,39,40,41,42,43,44,45]. Characteristics of included studies are presented in Table 1. All included studies were published from April 2021, mostly being research carried out in Europe (13 out 23). The sample size amongst the studies ranged between 74 and 3475 participants (median, 360), with a proportion of smokers between 4.2% and 40.8% (median, 26.0%). All reports assessed the impact of cigarette smoking, while in Yamamoto et al. users of heat-not-burn (HNB) tobacco products were also included [31]. Sixteen studies enrolled healthcare workers (HCW), two general population, and the others recruited patients with multiple sclerosis, cancer, inflammatory bowel disease, and obesity. Another report did not specify the enrolled population. Across the included reports, authors measured the humoral response to different available COVID-19 vaccines: 15 included participants immunized with BNT162b2 mRNA vaccine. in three CoronaVac was used, while the others presented various combination of BNT162b2 with ChAdOx1, mRNA-1273, mRNA-1273 and ChAdOx1, or BBIBP-CorV. Collection of a blood sample by venepuncture was performed at different time points across the reports, ranging from around 21 days to six months after the completion of the vaccination cycle. The studies included in the review analysis reflected a variety of serology tests for the research of IgG that bind the SARS-CoV-2 spike (S) protein receptor-binding domain (RBD), with specific positivity cutoff.

In 17 out of 23 studies, current smokers showed significant lower antibody titre, and in a few reports, highlighted a more rapid lowering of the vaccine-elicited IgG compared with nonsmokers [19,20,26,27,28,29,30,32,34,36,37,38,39,40,41,43,44]. In particular, accelerated antibody decline was reported in the prospective assessments by Ferrara et al. [20], Zhang et al. [27], and Malavazos et al. [30]. Of note, magnitude and timing of smoking-attributable lower antibody levels varied greatly across these studies, according to the type of serological test used and unit of measurement, the time since vaccination, and the analysis and adjustment performed. In all but two reports, the smoking time and quantity were not assessed. Indeed, duration of smoking or number of cigarettes per day did not predict the effect of smoking on the IgG titre in Nomura (*b*) et al. [37]; while in Yamamoto et al., smokers consuming 11 or more cigarettes per day showed a greater reduction in IgG than those consuming less than 11 cigarettes per day [31].

Regarding the studies that did not find a relationship between exposure to smoking and COVID-19 vaccine response, current smokers tended to have predominant lower antispike IgG levels than the past and never smoker groups, but the difference was not statistically significant in two reports [25,26]. Similarly, smoking status did not correlate with titres of IgG against the spike protein induced by BNT162b2 mRNA COVID-19 vaccine in the study of Modenese et al. [43], or those induced by either BNT162b2 or BBIBP-CorV COVID-19 vaccine in Alqassieh et al. [36]. In both the studies by Kato et al., which enrolled seven and five current smokers, respectively, there was no significant association between the titre of IgG against the spike protein induced by the vaccine and smoking habit [32,34].

In Yamamoto et al., HNB tobacco product users and dual users showed lowered geometric mean titres, but the differences from never smokers were not statistically significant, although the reduction reached statistical significance by combining the two categories of HNB tobacco users [31].

A complete overview of the studies’ findings is presented in Table 1.

The assessment with the GRADE approach found that quality of studies was moderate-to-high, and confirmed that the quality of the body of found evidence is acceptable for assessing the impact of smoking on humoral response to COVID-19 vaccines (Supplementary Materials, Table S2).

## 4. Discussion

To our knowledge, the present rapid systematic review is the first to show the impact of smoking on postvaccination antibody titres in relation to the use of cigarettes and HNB tobacco products. The vast majority of the current body of evidence suggests that smoking has a negative impact on the humoral response to COVID-19 vaccines, with both potential lower response and more rapid lowering of the vaccine-elicited IgG titres. However, the literature available so far does not allow us to firmly ascertain whether the effect is related to duration of smoking or number of cigarettes smoked per day [20,31,38].

The negative effects played by smoking on the immune system seem to be determined by several mechanisms that influence both innate and adaptive immunity. Regarding the first, certain studies have indicated a direct effect of smoking on alterations in immune cell counts (including monocytes, macrophages, dendritic cells, and lymphocytes), but the effect of the complex mixture of tobacco chemicals varies depending on the individual smoking habits, as well as several subsets of cells explored in different studies. Previous reports have also showed that smoking induced inflammatory cytokines and chemokines. Similarly, consistent animal and human studies have observed that cigarette smoking induces chronic inflammation and downregulates CD4+ T cells and B cells. In cigarettes smokers, the T cells also exhibit differences in proliferation response, indicating defective adaptive immunity responses. Furthermore, analyses of Ig revealed a decreased production of IgA, IgG, and IgM associated with smoking [5,20,46,47,48,49].

Across the found body of evidence, the pathophysiologic bases for the impact of active or ever smoking on the humoral response to COVID-19 vaccines have not been entirely suggested. Linardou et al. speculated that the smoking-attributable immunosuppressive effect is mediated by direct effects on T cells and the dendritic-cell system, which impairs host response to vaccination [38,48,49,50]. A recently published study from the VASCO project, an ongoing broad Italian study on the response to BNT162b2 mRNA COVID-19 vaccine in HCWs [16,17,18,20], underlined that cigarette smoking affects the ability to form memory cells that are critical to the maintenance of the protective immune response induced by vaccines, as well as smoking being associated with increased monocyte–macrophages counts, which may influence the clearance of circulating antibodies, whose half-life is of average 3–4 weeks (depending on IgG isotype and attributes) [18,20]. In Yamamoto, a lower decrease in vaccine-induced IgG levels was also seen in users of HNB tobacco products (compared with never smokers), although to a lesser extent than that associated with cigarette smoking. Authors attributed their findings to effects of nicotine—which is contained within HNB tobacco products at the same amounts as conventional cigarettes [51,52]—that might inhibit antibody-forming cell response, impair antigen-mediated signalling in T cells, and induce T-cell anergy [31,48].

Although a few analyses have separately described whether smoking affects the humoral response to vaccines or the antibody maintenance, the vast majority of the retrieved studies only investigated the IgG titres on defined time points, and we were unable to classify findings as reduced response or more rapid decay. Further research should investigate this important aspect of the overall impact of smoking on immunological response and maintenance [20]. It is worth also mentioning that some studies included in this review did not detect a correlation between smoking status and postvaccination IgG titres. However, most of these reports include a very low sample size and/or proportion of smokers [25,32,34,43], or examined the antibody levels in the early weeks after the completion of vaccination cycle [25,32,43], making it difficult to appreciate possible differences between smokers and nonsmokers [20].

Understanding possible factors that might influence the interindividual variability in vaccine response is important to ensure an effective response to vaccines [16,17,18,20,53]. While the mechanisms by which smoking impairs the humoral responses to COVID-19 vaccines deserve further research, our rapid review evidences the adverse effect of tobacco product use against immunogenicity to COVID-19 vaccination. Furthermore, some population surveys found more negative attitudes toward vaccination and unwillingness to vaccinate against COVID-19 amongst smokers compared with never and former smokers [54]. All together, these results advocate targeted policies to promote tailored health promotion initiatives tending to increase risk perception and ensure appropriate protection measures to be taken to avoid the infection and its consequences. Indeed, smoking cessation should be encouraged not only for prevention of the well-known smoking attributable diseases—namely cancer, respiratory illness, cardiovascular disease, etc.—but also due to the impact of smoking on immune function.

Of note, this study adds interesting insights on the current vivid research on the relationship between smoking and SARS-CoV-2/COVID-19. Indeed, research about the effects of active smoking on both infection and disease is still controversial, with some studies highlighting a lower proportion of positive SARS-CoV-2 serologies among current smokers and opposite evidence identifying smoking as a possible risk factor for disease progression [55]. In this regard, more research is needed to investigate the secondary health consequences of the effect smoking on vaccines response, in terms of vaccine effectiveness and risks of infection and reinfection.

Some limitations must be considered in this review. First, it was as a rapid review, which, despite being systematic in nature, was limited in the number of surfed databases. However, the assessment of the literature was in line with the minimum requirements (at least two databases) set by the PRISMA guidelines, and the review was conducted in accordance with WHO guidelines on rapid reviews [21,22]. Additionally, despite medRxiv being a preprint platform, without a peer-review process, it collects and publishes scientific reports on a very fast track, allowing us to consider the most updated available evidence [56]. In our view, this represents an important strength, especially considering the large number of studies conducted daily on COVID-19 [15,16,17]. Moreover, grey literature (webpages of international health authorities) was also consulted in order to collect and analyse all the available evidence. Nevertheless, at the time of study, evidence about this topic is still relatively sparse and the literature so far available does not allow us to consider the impact of possible aspects of smoking exposure (including duration of smoking, number of cigarettes per day, passive smoking exposure) and their impact on humoral response to COVID-19 vaccines, allowing us to only draw preliminary conclusions. For these reasons, and due to limited quality of data and reporting, these findings should be interpreted with caution, and require further exploration in studies specifically designed to examine the association between smoking and COVID-19 vaccine response [20,56]. Indeed, most of the found evidence relied on self-reported smoking status as part of wider investigation, and thus failed to assess and correct for possible sources of residual confounding [20,55]. Similarly, it is also worth mentioning that humoral response to vaccines could be affected by factors other than smoking exposure, including age, comorbidities and medication history of the vaccinees, number of vaccine doses. However, the use of multivariate analyses in most of the retrieved studies ensured the possibility of adjusting for well-known predictors of vaccine response, allowing us to estimate the independent effect of smoking. Of course, analyses of confounders will be always limited by the limited knowledge of all the “unknown” factors that can influence vaccine-elicited IgG kinetic. Again, the results presented here are time-limited to COVID-19 vaccines. However, the present research is the first to synthetize epidemiological studies on the impact of smoking on postvaccination antibody titres during the ongoing massive COVID-19 vaccination programs worldwide, providing important insights for public health and policymakers. Another limitation due to the design of a rapid systematic review is the absence of a registered protocol, which might have delayed study conduction and dissemination.

## 5. Conclusions

This rapid systematic review reveals that active smoking has a negative impact on the humoral response to COVID-19 vaccines. The findings suggest the need for tailored interventions directed towards smokers to ensure appropriate protection measures to be taken to avoid SARS-CoV-2 infection and COVID-19 consequences. This review also informs policymakers on how to draw specific actions to tackle health inequalities between smokers and nonsmokers, while also considering that smoking is closely linked to socioeconomically deprived populations who are at higher risk for health problems.

## Figures and Tables

**Table 1:** Characteristics and main findings of studies included in the systematic review.

First Author (and Year)	Country	Population Type and Numerosity	Male (%)	Mean or Median Age	Smokers (%)	COVID-19 Vaccine	Median Time Since Vaccination (in Days)	Serologic Test	Main Findings
Ferrara (2022) [20]	Italy	Healthcare workers, 162	42.0	42.5	34.8	BNT162b2	60	CLIA with reactivity cutoff equal to or greater than 1.0 AU/mL. Sensitivity of 100% (95%CI: 99.9–100) and specificity of 99.6% (95%CI: 98.7–100).	In the study of the dynamics of antibody response to COVID-19 vaccine after 6 months, at the 60-day serology, a difference in vaccine-induced IgG titre was seen, with median antibody titres of, respectively, 211.80 (IQR 149.80–465.50) and 487.50 (IQR 308.45–791.65) AU/mL (*p*-value = 0.002). In the multivariate regression model, the β coefficient was equal to −335.62 (95%CI: −557.41–−113.83; *p* = 0.004) for active smoking. No other differences were seen in other sample timings (30 days, and 3–6 months).
Gümüş (2021) [25]	Turkey	Healthcare workers, 94	54.3	41	36.2	CoronaVac	21	CLIA with reactivity cutoff equal to or greater than 1.1. Sensitivity: NR; specificity: NR.	Seropositivity was predominantly detected nonsmokers, but the difference was not statistically significant (64.1%, *p*-value = 0.999)
Ikezaki (2021) [26]	Japan	Healthcare workers, 373	20.1	42	5.9	BNT162b2	185	CMIA with positivity cutoff equal to or greater than 50 AU/mL. Sensitivity of 98.3% (95%CI: 90.6–100) and specificity 99.5% (95%CI: 97.1–100).	The current smoker group tended to have lower antispike IgG levels than the past and never-smoker groups, but the difference was not statistically significant.
Zhang (2021) [27]	China	NR, 164	23.2	34	6.7	CoronaVac	14, 42, and 90	SARS-CoV-2 S-RBD protein microarray. COI: NR. Sensitivity: NR; specificity: NR.	Compared to nonsmokers, the levels of neutralizing antibodies in smokers remained low throughout the period of testing. Notably, the median IgG titres in the smoking group was 1.40-, 1.32-, or 3.00-fold lower than that of nonsmoking group on day 14, 42, or 90, respectively.
Pitzalis (2021) [28]	Italy	Multiple Sclerosis patients, 658	26.9	48,8	28.6	BNT162b2	30	ECLIA with reactivity COI equal to or greater than 1.0. Sensitivity of 99.5% (95%CI: 97.0–100) and specificity of 99.8% (95%CI: 99.7–99.9).	There was a reduced anti-S antibodies production in smokers (median = 719 U/mL) compared to nonsmokers (median = 1054 U/mL) (*p*-value < 0.001).
Herzberg (2021) [29]	Germany	Healthcare workers, 562	22.8	43.5	26.0	BNT162b2 (two doses) or ChAdOx1 (one dose)	21–90	ELISA with positivity ratio equal to or greater than 1.1. Sensitivity of 100% (95%CI: 91.6–100) and specificity of 97.7% (95%CI: 91.9–99.6).	A negative effect of current smoking on antibody response was observed at linear regression for anti-SARS-CoV-2 antibody ratio: estimate −0.41 (95%CI, −0.70–−0.12; *p*-value = 0.006).
Malavazos (2021) [30]	Italy	Patients with obesity, 1060	38.0	41.4	15.8	BNT162b2	T_0_ and day 21 after the first; and within 30–40 and 90–100 days after the second dose	CLIA with positivity threshold equal to or greater than 33.8 BAU/mL. Sensitivity of 98.7% (95%CI: 94.5–99.6) and specificity of 99.5% (95%CI: 99.0–99.7).	Smoking was associated with drops in IgG-TrimericS levels at three months after the second dose (absolute variation in IgG levels starting from one month after the second dose) at univariate (*p*-value = 0.03) and multivariate linear regression analyses (*p*-value = 0.04).
Yamamoto (2021) [31]	Japan	Healthcare workers, 3457	38.0	41	6.1	BNT162b2	64	CLEIA with positivity threshold equal to or greater than 10 SU/mL. Sensitivity of 98.3% and specificity of 99.6%.	Of 212 current smokers, 53% used HNB tobacco products. Current smokers using any tobacco product had lower antibody titres (GMT, 101; ratio of mean, 0.85 [95%CI: 0.77–0.93]) compared with never smokers. Exclusive cigarette smokers had significantly lower GMT than never smokers (GMT, 119 versus 99; ratio of means, 0.81 [95%CI: 0.71–0.92]). Exclusive HNB tobacco product users and dual users also showed similarly lowered GMT (103 and 108, respectively), although the differences from never smokers were not statistically significant (ratio of means, 0.87 [95%CI: 0.74–1.02] and 0.91 [95%CI: 0.76–1.08], respectively). Combining the two categories of HNB tobacco users (*n* = 113), the reduction reached statistical significance (GMT, 105; ratio of mean, 0.88 [95%CI: 0.78–0.99]). Among daily cigarette smokers, those consuming 11 or more cigarettes per day showed a greater reduction in IgG titres than those consuming less than 11 cigarettes per day; GMTs (ratio of means) were 92 (0.77 [95%CI: 0.62–0.95]) and 104 (0.87 [95% CI: 0.76–1.00]), respectively.
Kato [a] (2021) [32]	Japan	Healthcare workers, 168	25.0	43	4.2	BNT162b2	14, 28 and 42 after the first dose	CLEIA with cut-off index equal to or greater than 1. Sensitivity of 100% (95%CI: 97.6–100) and specificity 100% (95%CI: 99.6–100).	There was no significant association between the titre of IgG against spike proteins induced by the vaccine and smoking habit (*p*-value = 0.44).
Nomura [a] (2021) [33]	Japan	Healthcare workers, 365	31.5	44	40.8	BNT162b2	183	ECLIA with reactivity COI equal to or greater than 1.0. Sensitivity of 99.5% (95%CI: 97.0–100) and specificity of 99.8% (95%CI: 99.7–99.9).	Smokers group: 149 ever smokers of which 90 current smokers. The age-adjusted median (IQR) antibody titres were −97 (−277 to 184) and 56 (−182 to 342) in ever-smokers and never smokers, respectively (*p*-value < 0.001); and −205 (−320 to 7) and −72 (−264 to 256) in current-smokers and never smokers, respectively (*p*-value = 0.03). For age-adjusted median antibody titres, no significant sex differences were observed in the ever-smoker and never-smoker groups. However, both the male and female groups showed significant differences by smoking status in age-adjusted median antibody titres. No significant differences in the median rate of change in antibody titres by smoking status were observed in the male and female groups. Both the ever-smoker and never-smoker groups showed significant sex differences in the median rate of change in antibody titres.
Kato [b] (2021) [34]	Japan	Healthcare workers, 98	42.4	43	5.6	BNT162b2	180	CLEIA with cutoff index equal to or greater than 1. Sensitivity of 100% (95%CI: 97.6–100) and specificity 100% (95%CI: 99.6–100).	The titre of IgG against spike proteins induced by the vaccine did not correlate with smoking status.
Uysal (2021) [35]	Turkey	Healthcare workers, 314	42.4	40	32.5	CoronaVac	30	ECLIA with reactivity COI equal to or greater than 1.0 and the highest antibody value was measured as 250 U/mL by the device. Sensitivity of 99.5% (95%CI: 97.0–100) and specificity of 99.8% (95%CI: 99.7–99.9).	When the smoking habit and antibody response were compared, 40% of those with an antibody titre of 1–125 U/mL had a history of smoking, while this rate was decreased down to 24.7 in the group with 126–250 U/mL, and to 27.5% in participants with seropositivity of more than 250 U/mL: thus, 72.5% of those with an antibody titre of more than 250 U/mL were nonsmokers (*p*-value = 0.03).
Alqassieh (2021) [36]	Jordan	General population, 288	65.6	NR	31.6	BNT162b2 or BBIBP-CorV	42	ELFA with positivity cutoff index equal to or greater than 1. Sensitivity: NR; specificity: NR.	No significant differences were found between the two groups in terms of smoking habit (*p*-value = 0.351), with either BNT162b2 and BBIBP-CorV COVID-19 vaccine
Nomura [b] (2021) [37]	Japan	Healthcare workers, 378	32.5	44	40.7	BNT162b2	90	ECLIA with reactivity COI equal to or greater than 1.0. Sensitivity of 99.5% (95%CI: 97.0–100) and specificity of 99.8% (95%CI: 99.7–99.9).	Smokers: 49 current smokers. In both the male and female groups, age-adjusted median antibody titres were significantly lower in ever smokers than in never smokers; age-adjusted median antibody titres (IQR) in men were −246 U/mL (−398 to 65) and 49 U/mL (−186 to 621) in ever smokers and never smokers, respectively, while those in women were −140 U/mL (−304 to 217) and 95 U/mL (−151 to 503) in ever smokers and never smokers, respectively. Compared with never smokers, median IgG were −271 (−475 to 33; *p*-value < 0.0001) for current smokers, and −162 (−332 to 285; *p*-value = 0.0019) for exsmokers. Antibody titres were significantly lower in current smokers than in exsmokers (*p*-value = 0.019). The number of cigarettes per day did not influence the antibody titres. In both the ever smoker and never smoker groups, no significant sex differences in age-adjusted median antibody titres were observed. Given that the smoking rates in the male and female groups were 61.0% and 31.0%, respectively, these results suggest that the sex difference in antibody titres strongly reflects sex differences in smoking, rather than biological sex differences.
Linardou (2021) [38]	Greece	Cancer patients, 189	46.0	NR	30.6	BNT162b2, mRNA-1273, or ChAdOx1	30	CLIA with positivity threshold equal to or greater than 33.8 BAU/mL. Sensitivity of 98.7% (95%CI: 94.5–99.6) and specificity of 99.5% (95%CI: 99.0–99.7).	A significant association was identified between IgG titres and smoking status (Kruskal–Wallis *p*-value = 0.017). Post hoc analysis revealed that never smokers had significantly higher antibody titres compared with current smokers (median value: 632 vs. 409.5, Wilcoxon rank-sum *p*-value = 0.006).
Tsatsakis (2021) [19]	Greece	Healthcare workers, 517	33.7	47.7	34.4	BNT162b2	60	ELISA with positivity ratio equal to or greater than 1. Sensitivity of 97.3% (95%CI: 90.8–99.3) and specificity of 100% (95%CI: 96.0–100).	Nonsmokers had higher titres than smokers: 4.48 (±2.79 SD) and 3.80 (±2.64 SD), respectively; *p*-value = 0.003). No significance at multivariate linear regression analysis of antibody titre sampling postvaccination was found.
Moncunill (2021) [39]	Spain	Healthcare workers, 360	26.1	43.2	22.2	BNT162b2 or mRNA-1273	Up to 20 post vaccination	Quantitative suspension array technology with sensitivity of 95.8% and specificity of 100%. COI: NR.	Smoking was associated with significantly lower IgG S levels (62.5%; 95%CI 5.6–85.1; *p*-value = 0.038) after one (>7 days) and two doses of mRNA vaccines (12–19 days postvaccine). Being a smoker was also associated with 42.8% (95%CI 59.5–19.2; *p*-value = 0.002) lower plasma-neutralizing capacity.
Parthymou (2021) [40]	Greece	General population, 712	37.6	50.8	34.4	BNT162b2	~ 90	ECLIA with reactivity COI equal to or greater than 1.0. Sensitivity of 99.5% (95%CI: 97.0–100) and specificity of 99.8% (95%CI: 99.7–99.9).	Multivariate linear regression analysis revealed a negative association between smoking and antibody titre: β of −0.1097 (95%CI −0.173–−0.04567; *p*-value = 0.0008). The mean antibody titre of smokers 988 (±781.4 SD) versus 731.2 (±603.9 SD) in nonsmokers.
Michos (2021) [41]	Greece	Healthcare workers, 264	20.1	45.4	25.8	BNT162b2	30	ECLIA with reactivity COI equal to or greater than 1.0. Sensitivity of 99.5% (95%CI: 97.0–100) and specificity of 99.8% (95%CI: 99.7–99.9).	Smokers had a statistically significant lower antibody response for TAbs-RBD and NAbs-RBD after both the first (assessed after 20 days from the vaccination) and second vaccine doses (*p*-value = 0.033, *p*-value = 0.015, *p*-value =0.041, *p*-value = 0.002, respectively). At linear regression analysis, after the first vaccine dose, a statistically significant negative association of TAbs-RBD was detected for smoking status (*p*-value = 0.012). After the second vaccine dose, a statistically significant negative association of TAbs-RBD was detected for age smoking status (*p*-value = 0.011).
Lombardi (2021) [42]	Italy	Healthcare workers, 3475	28.8	NR	23.1	BNT162b2	28	ECLIA with reactivity COI equal to or greater than 1.0. Sensitivity of 99.5% (95%CI: 97.0–100) and specificity of 99.8% (95%CI: 99.7–99.9).	Smokers showed lower median titres than never smokers.
Modenese (2021) [43]	Italy	Healthcare workers, 74	19.9	48.4	23.0	BNT162b2	28	CLIA with reactivity cutoff equal to or greater than 1.0 AU/mL. Sensitivity of 100% (95%CI: 99.9–100) and specificity of 99.6% (95%CI: 98.7–100).	Smoking habit did not significantly affect the IgG titre (*p*-value = 0.55)
Watanabe (2021) [44]	Italy	Healthcare workers, 86	39.5	29	31.7	BNT162b2	30	ECLIA with reactivity COI equal to or greater than 1.0. Sensitivity of 99.5% (95%CI: 97.0–100) and specificity of 99.8% (95%CI: 99.7–99.9).	Smokers had lower levels compared to nonsmokers [1099 (±1350 SD) vs. 1921 U/mL (±1375 SD), *p*-value = 0.007], at multivariate linear regression β coefficient was −698.28 (−1228.87 to −167.69) for current smokers (*p*-value = 0.011)
Kennedy (2021) [45]	United Kingdom	Inflammatory bowel disease patients, 1293	50.7	43.8	8.3	BNT162b2 or ChAdOx1	21–70	ECLIA with reactivity COI equal to or greater than 1.0. Sensitivity of 99.5% (95%CI: 97.0–100) and specificity of 99.8% (95%CI: 99.7–99.9). Positivity threshold fixed at 0.25-fold COI for patients with prior infection; of 0.12-fold for those with no evidence of prior infection.	Current smoking was independently associated with lower anti-SARS-CoV-2 antibody concentrations in subjects who received either vaccine. Fold change for both vaccines 0.53 (95%CI, 0.36−0.74; *p*-value < 0.001); for BNT162b2 alone 0.52 (95%CI, 0.31−0.86; *p*-value = 0.011); for ChAdOx1 alone 0.55 (95%CI, 0.36−0.84; *p*-value = 0.006).

Abbreviations: COVID-19, coronavirus disease 2019; SARS-CoV-2, severe acute respiratory syndrome-coronavirus 2; HNB, heat-not-burn tobacco products; CLIA, chemiluminescent immunoassay; CMIA, chemiluminescent microparticle immunoassay; ECLIA, electrochemiluminescence immunoassay; CLEIA, chemiluminescence enzyme immunoassay; ELISA, enzyme-linked immunosorbent assay; ELFA, enzyme-linked fluorescent assay; COI, cutoff index; IgG, immunoglobulins G; AU, antibody unit; BAU, binding antibody unit; GMT, geometric mean titre; IQR, interquartile range; SD, standard deviation; 95% CI, 95% confidence interval; NR, not reported.

## Data Availability

Not applicable.

## Supplementary Materials

The following supporting information can be downloaded at: https://www.mdpi.com/article/10.3390/vaccines10020303/s1, Table S1: List of full-text reports not accepted for inclusion in the rapid systematic review with exclusion reasons; Table S2: Summary of GRADE’s approach for the quality rating of the body of evidence.
