# Supplementary material for: The Effect of Smoking on Humoral Response to COVID-19 Vaccines: A Systematic Review of Epidemiological Studies
Source: Vaccines (Basel). 2022 Feb 16;10(2):303. doi: 10.3390/vaccines10020303 (PMC8880575; doi:10.3390/vaccines10020303)
Supplement: Supplementary file 1 [file vaccines-10-00303-s001.zip › vaccines-1572866-supplementary.pdf]

Supplementary material

Effect of smoking on the immunological response to COVID-19 vaccines: A rapid systematic review

Table S1. **List of full-text reports not accepted for inclusion in the rapid systematic review with exclusion reasons**

**Exclusion reason**	**Citation**
No data on humoral response after COVID-19 vaccination for smokers	Buttiron Webber T, Provinciali N, Musso M, et al. Predictors of poor seroconversion and adverse events to SARS-CoV-2 mRNA BNT162b2 vaccine in cancer patients on active treatment. Eur J Cancer. 2021;159:105-112. doi: 10.1016/j.ejca.2021.09.030.
	Zhao Z, Salerno S, Shi X, Lee S, Mukherjee B, Fritsche LG. Understanding the Patterns of Serological Testing for COVID-19 Pre- and Post-Vaccination Rollout in Michigan. Journal of Clinical Medicine. 2021; 10(19):4341. https://doi.org/10.3390/jcm10194341
No stratification according smoking status/habit	Levy I, Wieder-Finesod A, Litchevsky V, et al. Immunogenicity and safety of the BNT162b2 mRNA COVID-19 vaccine in people living with HIV-1 Clin Microbiol Infect. 2021;27(12):1851-1855. doi: 10.1016/j.cmi.2021.07.031.
	Firinu D, Perra A, Campagna M, Littera R, Meloni F, Sedda F, Conti M, Costanzo G, Erbi M, Usai G, Locci C, Carta MG, Cappai R, Orrù G, Del Giacco S, Coghe F, Chessa L. Evaluation of Antibody Response to Heterologous Prime–Boost Vaccination with ChAdOx1 nCoV-19 and BNT162b2: An Observational Study. Vaccines. 2021; 9(12):1478. https://doi.org/10.3390/vaccines9121478
	Madhi SA, Koen AL, Izu A, et al. Safety and immunogenicity of the ChAdOx1 nCoV-19 (AZD1222) vaccine against SARS-CoV-2 in people living with and without HIV in South Africa: an interim analysis of a randomised, double-blind, placebo-controlled, phase 1B/2A trial. The Lancet HIV 2021;8(9): E568-E580
	Arulkumaran N, Snow TAC, Kulkarni A, et al. Sex differences in immunological responses to COVID-19: a cross-sectional analysis of a single-centre cohort. Br J Anaesth. 2021;127(2):e75-e78. doi: 10.1016/j.bja.2021.05.013.
Study design	Ferrari et al. Systematic evaluation of the tolerability of two doses of the Pfizer-BioNTech COVID-19 vaccine (BNT162b2) in a diverse cohort of people with HIV (PWH) (Conference abstract)
	Della Pia A, Youn Kim G, Ahn J, et al. Production of Anti-Spike Antibodies in Response to COVID Vaccine in Lymphoma Patients. Blood 2021; 138 (S1): 1347. doi: https://doi.org/10.1182/blood-2021-151367
	Colaneri M, De Filippo M, Licari A, et al. COVID vaccination and asthma exacerbation: might there be a link? Int J Infect Dis. 2021;112:243-246. doi: 10.1016/j.ijid.2021.09.026.
Language	Şenol Akar Ş, Akçalı s, Özkaya Y, et al. [Factors Affecting Side Effects, Seroconversion Rates and Antibody Response After Inactivated SARS-CoV-2 Vaccination in Healthcare Workers] Mikrobiyol Bul. 2021;55(4):519-538. doi: 10.5578/mb.20219705. [Article in Turkish]

Table S2. **Summary of GRADE's approach for the quality rating of the body of evidence**

**Research question**<br>Humoral immunogenicity of COVID-19 vaccination in smokers and non-smokers: a rapid systematic review							
**Body of evidence**<br>23 observational researches							
23 studies published in peer-reviewed journals				6 preprints			
**Risk of Bias**	**Inconsistency**	**Indirectness**	**Imprecision**	**Risk of Bias**	**Inconsistency**	**Indirectness**	**Imprecision**
No serious	No serious	No serious	No serious	No serious	No serious	No serious	No serious
Other considerations				Other considerations			
Lack of sampling strategy for smoker sub-group numerosity. Scarce methodological information on type and characteristics of serologic tests.				Lack of sampling strategy for smoker sub-group numerosity, low proportion of smokers. Lack of methodological information on type and characteristics of serologic tests. No satisfactory statistical adjustment.			
Quality of the body of evidence:<br><br>Moderate (three plus: ⊕⊕⊕○)				Quality of the body of evidence:<br><br>Low (two plus: ⊕⊕○○)			

The quality of evidence was assessed following the Grading of Recommendation Assessment, Development and Evaluation (GRADE) guidelines, available at: Balshema H, Helfanda M, Schünemann HJ, et al. GRADE guidelines: 3. Rating the quality of evidence. J Clin Epidemiol. 2011;64:401-406.

PRISMA 2020 Checklist

Section and Topic	Item #	Checklist item	Location where item is reported
**TITLE**			
Title	1	Identify the report as a systematic review.	Page 1
**ABSTRACT**			
Abstract	2	See the PRISMA 2020 for Abstracts checklist.	Page 2
**INTRODUCTION**			
Rationale	3	Describe the rationale for the review in the context of existing knowledge.	Page 3
Objectives	4	Provide an explicit statement of the objective(s) or question(s) the review addresses.	Page 4
**METHODS**			
Eligibility criteria	5	Specify the inclusion and exclusion criteria for the review and how studies were grouped for the syntheses.	Page 5
Information sources	6	Specify all databases, registers, websites, organisations, reference lists and other sources searched or consulted to identify studies. Specify the date when each source was last searched or consulted.	Page 4
Search strategy	7	Present the full search strategies for all databases, registers and websites, including any filters and limits used.	Page 4
Selection process	8	Specify the methods used to decide whether a study met the inclusion criteria of the review, including how many reviewers screened each record and each report retrieved, whether they worked independently, and if applicable, details of automation tools used in the process.	Page 4-5
Data collection process	9	Specify the methods used to collect data from reports, including how many reviewers collected data from each report, whether they worked independently, any processes for obtaining or confirming data from study investigators, and if applicable, details of automation tools used in the process.	Page 4
Data items	10a	List and define all outcomes for which data were sought. Specify whether all results that were compatible with each outcome domain in each study were sought (e.g. for all measures, time points, analyses), and if not, the methods used to decide which results to collect.	Table 1
	10b	List and define all other variables for which data were sought (e.g. participant and intervention characteristics, funding sources). Describe any assumptions made about any missing or unclear information.	Table 1
Study risk of bias assessment	11	Specify the methods used to assess risk of bias in the included studies, including details of the tool(s) used, how many reviewers assessed each study and whether they worked independently, and if applicable, details of automation tools used in the process.	NA
Effect measures	12	Specify for each outcome the effect measure(s) (e.g. risk ratio, mean difference) used in the synthesis or presentation of results.	Table 1
Synthesis methods	13a	Describe the processes used to decide which studies were eligible for each synthesis (e.g. tabulating the study intervention characteristics and comparing against the planned groups for each synthesis (item #5)).	Page 5
	13b	Describe any methods required to prepare the data for presentation or synthesis, such as handling of missing summary statistics, or data conversions.	Page 5
	13c	Describe any methods used to tabulate or visually display results of individual studies and syntheses.	Page 5
	13d	Describe any methods used to synthesize results and provide a rationale for the choice(s). If meta-analysis was performed, describe the model(s), method(s) to identify the presence and extent of statistical heterogeneity, and software package(s) used.	NA
	13e	Describe any methods used to explore possible causes of heterogeneity among study results (e.g. subgroup analysis, meta-regression).	NA

Section and Topic	Item #	Checklist item	Location where item is reported
	13f	Describe any sensitivity analyses conducted to assess robustness of the synthesized results.	NA
Reporting bias assessment	14	Describe any methods used to assess risk of bias due to missing results in a synthesis (arising from reporting biases).	Page 5
Certainty assessment	15	Describe any methods used to assess certainty (or confidence) in the body of evidence for an outcome.	NA
**RESULTS**			
Study selection	16a	Describe the results of the search and selection process, from the number of records identified in the search to the number of studies included in the review, ideally using a flow diagram.	Page 5
	16b	Cite studies that might appear to meet the inclusion criteria, but which were excluded, and explain why they were excluded.	NA
Study characteristics	17	Cite each included study and present its characteristics.	Page 5
Risk of bias in studies	18	Present assessments of risk of bias for each included study.	Page 6
Results of individual studies	19	For all outcomes, present, for each study: (a) summary statistics for each group (where appropriate) and (b) an effect estimate and its precision (e.g. confidence/credible interval), ideally using structured tables or plots.	NA
Results of syntheses	20a	For each synthesis, briefly summarise the characteristics and risk of bias among contributing studies.	Appendix
	20b	Present results of all statistical syntheses conducted. If meta-analysis was done, present for each the summary estimate and its precision (e.g. confidence/credible interval) and measures of statistical heterogeneity. If comparing groups, describe the direction of the effect.	NA
	20c	Present results of all investigations of possible causes of heterogeneity among study results.	Page 6
	20d	Present results of all sensitivity analyses conducted to assess the robustness of the synthesized results.	Page 6
Reporting biases	21	Present assessments of risk of bias due to missing results (arising from reporting biases) for each synthesis assessed.	Page 6
Certainty of evidence	22	Present assessments of certainty (or confidence) in the body of evidence for each outcome assessed.	NA
**DISCUSSION**			
Discussion	23a	Provide a general interpretation of the results in the context of other evidence.	Page 6-8
	23b	Discuss any limitations of the evidence included in the review.	Page 6-8
	23c	Discuss any limitations of the review processes used.	Page 8
	23d	Discuss implications of the results for practice, policy, and future research.	Page 7-8
**OTHER INFORMATION**			
Registration and protocol	24a	Provide registration information for the review, including register name and registration number, or state that the review was not registered.	Page 8
	24b	Indicate where the review protocol can be accessed, or state that a protocol was not prepared.	NA
	24c	Describe and explain any amendments to information provided at registration or in the protocol.	NA
Support	25	Describe sources of financial or non-financial support for the review, and the role of the funders or sponsors in the review.	Page 8

Section and Topic	Item #	Checklist item	Location where item is reported
Competing interests	26	Declare any competing interests of review authors.	Page 8-9
Availability of data, code and other materials	27	Report which of the following are publicly available and where they can be found: template data collection forms; data extracted from included studies; data used for all analyses; analytic code; any other materials used in the review.	NA
